# Spatial effects of mosquito bednets on child mortality

**DOI:** 10.1186/1471-2458-8-356

**Published:** 2008-10-14

**Authors:** Laura Gosoniu, Penelope Vounatsou, Adriana Tami, Rose Nathan, Hajo Grundmann, Christian Lengeler

**Affiliations:** 1 Swiss Tropical Institute, Basel, Switzerland; 2 Royal Tropical Institute, Biomedical Research, Amsterdam, the Netherlands; 3 Ifakara Health Research and Development Center, Ifakara, Tanzania; 4 Centre for Infectious Diseases Epidemiology, National Institute for Public Health and the Environment, Bilthoven, the Netherlands

## Abstract

**Background:**

Insecticide treated nets (ITN) have been proven to be an effective tool in reducing the burden of malaria. Few randomized clinical trials examined the spatial effect of ITNs on child mortality at a high coverage level, hence it is essential to better understand these effects in real-life situation with varying levels of coverage. We analyzed for the first time data from a large follow-up study in an area of high perennial malaria transmission in southern Tanzania to describe the spatial effects of bednets on all-cause child mortality.

**Methods:**

The study was carried out between October 2001 and September 2003 in 25 villages in Kilombero Valley, southern Tanzania. Bayesian geostatistical models were fitted to assess the effect of different bednet density measures on child mortality adjusting for possible confounders.

**Results:**

In the multivariate model addressing potential confounding, the only measure significantly associated with child mortality was the bed net density at household level; we failed to observe additional community effect benefit from bed net coverage in the community.

**Conclusion:**

In this multiyear, 25 village assessment, despite substantial known inadequate insecticide-treatment for bed nets, the density of household bed net ownership was significantly associated with all cause child mortality reduction. The absence of community effect of bednets in our study area might be explained by (1) the small proportion of nets which are treated with insecticide, and (2) the relative homogeneity of coverage with nets in the area. To reduce malaria transmission for both users and non-users it is important to increase the ITNs and long-lasting nets coverage to at least the present untreated nets coverage.

## Background

*Plasmodium falciparum *malaria is a leading infectious disease, accounting for approximately 300 to 500 million clinical cases each year and causing over one million deaths, mostly in African children younger than 5 years. Insecticide treated nets (ITN) have been proven to be an effective tool in reducing the burden of malaria [1-3]. Numerous trials all over the world have shown that such nets can reduce child mortality in endemic areas in Africa by 17% and roughly halve the number of clinical malaria episodes [4]. These results were later confirmed under programme implementation [5,6]. It is well known that the use of ITNs provides significant individual protection, but direct and indirect effects on malaria transmission of treated and untreated nets on the wider community of bednet users and non-users are still little understood, despite some recent progresses [7]. Randomised trials in different malaria transmission regions examined the effect of ITNs on mortality of children without bednets. A study carried out in northern Ghana estimated that mortality risk in individuals without insecticide nets increased by 6.7% with every 100 m shift away from the nearest intervention compound [8]. In western Kenya households without ITNs but within 300 m of ITN villages received nearly full protection [9]. These results conflict with those found from studies in The Gambia which concluded that protection against malaria seen in children using ITN is due to personal rather than community effect [10-12]. A better understanding of these spatial effects in real-life situations is paramount for setting control targets, especially for understanding equity issues since these spatial effects mainly improve the situation of unprotected individuals, who are on average poorer. Moreover, the spatial effects of ITNs on non-bednet users in relation with the degree of density of bednets will indicate the type and level of bednet coverage that control programs need to achieve in order to maximize protection of non-bednet users. Here we present for the first time results for the spatial effects of mosquito nets in a "real-life" programme. One of the limitations of previous studies is that they used standard statistical methods which assume independence between observations. When these methods are applied to spatially correlated data, they underestimate the standard errors and thus overestimate the statistical significance of the covariates [13]. In this paper we analyzed data from a large follow-up study in a highly malaria endemic area in southern Tanzania. Making use of a demographic surveillance system (DSS) we tracked child mortality prospectively and assessed the relation between all-cause child mortality rates and the spatial effect of bednet density. To account for spatial clustering we fitted Bayesian geostatistical models with household-specific random effects. Models for geostatistical data introduce the spatial correlation in the covariance matrix of the household-specific random effects and model fit is based on Markov chain Monte Carlo methods (MCMC). MCMC estimation requires repeated inversions of the covariance matrix which, for large number of locations is computationally intensive and time consuming. To address this problem we propose a convolution model for the underlying spatial process which replaces large matrix inversion by the inversion of much smaller matrices.

## Methods

### Study area and population

**Figure 1:**
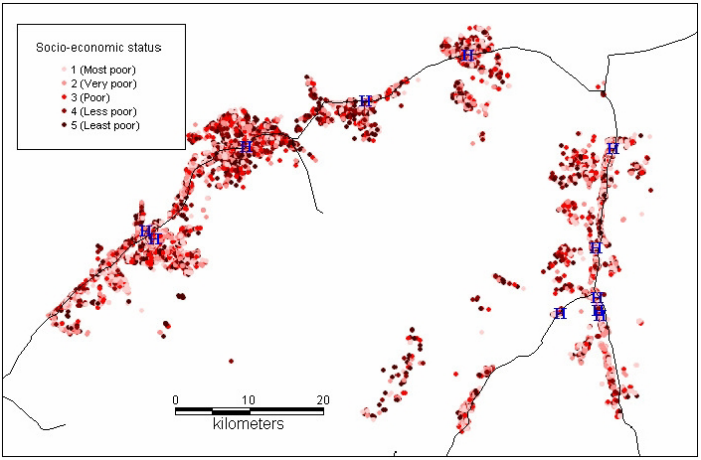
**Distribution of the DSS households according to their socio-economic status**. Socio-economic status of the DSS households: from light red to dark red: most poor, very poor, poor, less poor and least poor.

The study was carried out from October 2001 to September 2003 in the 25 villages covered by a demographic surveillance system (DSS) in the Kilombero Valley, southern Tanzania. The DSS updates every 4 months demographic information on a population of about 73, 000 people living in 12, 000 dispersed households (Figure 1) in two districts – Kilombero and Ulanga [14]. Most residents practice subsistence farming with rice and maize being the predominant crops. The climate is marked by a rainy season from November to May with annual rainfall ranging from 1200 to 1800 mm. Malaria is the foremost health problem, for both adults and children [15]. The prevailing malaria vectors in this region are *Anopheles gambiae *and *Anopheles funestus *with an estimated average entomological inoculation rate estimated of over 360 infective bites per person a year [16]. A large-scale social marketing programme of ITNs for malaria control has been running in this area since 1997 [6].

### Data collection

Mortality data were obtained prospectively and continuously over a two-year period from the DSS, which allowed us to register age and sex data, births and migrations in and out the study area. Exact procedures are described in [14].

An additional survey was carried out in the DSS population in 2002 to collect socio-economic information. The survey questionnaire included a list of household assets (e.g. bednet), housing characteristics (e.g. type of roofing material) and type of energy and light. Although information on ITNs ownership was also collected, we did not use these data in our analysis since it was shown [17] that in this area two-thirds of the nets that were reported as having been re-treated within the last 12 months had insufficient insecticide to be effective.

Households and health facilities were geolocated using a hand-held Global Positioning System (Garmin GPS 12, Garmin corp.) and Euclidean distances between houses and the health facilities were calculated. Oral informed consent was obtained from the heads of the households included in the study.

### Statistical analysis

Bednet density was defined as the number of bednets per person within a certain radius around each household. The following radii were chosen: 0 m (bednet coverage at household level), 50 m, 100 m, 150 m, 200 m, 300 m, 400 m, 500 m and 600 m.

A wealth index was calculated as a weighted sum of household assets. It has been shown that there is an inverse relationship between mortality and socio-economic status [18]; therefore the weights of the wealth index were obtained from the coeffcients of a negative binomial model which estimated the effect of assets on all-age mortality. The weight of asset *i *was calculated as $w_{i}=\frac{b_{i}}{\sqrt{\sum_{i}b_{i}^{2}}}$, where *b*_*i *_is the regression coeffcient corresponding to asset *i*. The wealth index was divided into quintiles corresponding to poorest, very poor, poor, less poor and least poor groups of the population.

Negative binomial models were fitted to assess the effect of different bednet density measures on child mortality after adjusting for possible confounders: sex, wealth index and distance to the nearest health facility, using STATA v. 9.0 (Stata Corporation, College Station, TX, USA).

To estimate the effect of bednet density on the mortality of children without nets we performed a similar analysis. In particular, we defined bednet density as above, considering as index households the ones without any bednet. We then fitted the negative binomial models adjusted for the above mentioned confounders.

The household mortality data are correlated in space since common environmental risk factors, proximity to breeding sites and socio-economic exposures may influence the mortality outcome similarly in households within the same geographical area. The independence assumption of the standard negative binomial models may result in overestimation of the significance of the bednet coverage covariate. To address this problem Bayesian geostatistical negative binomial models were fitted with household-level random effects. Spatial correlation was modeled by assuming that the random effects are distributed according to a multivariate normal distribution with variance-covariance matrix related to an exponential correlation function between household locations, i.e. *σ*^2 ^*exp *(*-d*_*ij*_*ρ*), where *d*_*ij *_is the Euclidean distance between households *i *and *j*, *σ*^2 ^is the geographic variability known as the sill and *ρ *is the rate of correlation decay. The distribution of random effect defines the so called Gaussian spatial process. Model fit requires the inversion of a covariance matrix with the same size as the sample size. Due to the large number of observations in our dataset, the estimation of model parameters becomes unstable and unfeasible. To overcome this problem we propose a model based on a convolution representation that is, we approximate the spatial random process by a weighted sum of a small number of stationary spatial processes. The size of the covariance matrix that needs to be inverted is then much smaller, therefore the method is computationally efficient. We employed Markov chain Monte Carlo simulation to estimate the model parameters. Further details on this modeling approach are given in the appendix. The analysis was implemented using a software written by the authors in FORTRAN 95 (Compaq Visual FORTRAN Professional 6.6.0) using standard numerical libraries (NAG, The Numerical Algorithm Group Ltd.).

## Results

A total number of 11, 134 children from 7, 403 households with children had information available on both geolocation and socio-economic covariates.

The pooled data revealed an overall all-age crude mortality rate of 9.5 per 1000 person-years and an overall child mortality of 26.2 per 1000 person-years with no difference between the two districts (*P *= 0.98 and *P *= 0.73, respectively).

The insecticide treatment status of the nets was difficult to ascertain, therefore the results reported in this section refer to bednets only, whether treated or not. The mean bednet density in Kilombero Valley was 270 nets per 1000 inhabitants. 10, 160 households (85%) had at least one bednet and the mean number of bednets per household was 1.64.

Table 1 shows the overall child mortality rates together with district-specific child mortality rates by sex, socio-economic status, distance to the nearest health facility and bednet density at household level. Since there were no significant differences between child mortality rates in Kilombero and Ulanga Districts, all further analysis was done by pooling the data of the two districts. Males had a slightly lower mortality rate than females, but sex was not significantly associated with childhood mortality rates (Incidence-Rate Ratios (*IRR*) = 0.90, *P *= 0.216). Similarly, socio-economic status was not significantly associated with child mortality (*P *= 0.124), but we could notice a trend for children from the relatively better off households to have a lower mortality rate than their poorer counterparts. No significant association was observed with distance to the nearest health facility, but children living ≥ 1 km away from the nearest health facility tended to have higher mortality rates than those living in close proximity.

**Table 1:** Overall and district-specific child mortality rates by sex, socio-economic status, distance to the nearest health facility and bednet density at household level

Explanatory variables	Number of children(%)	Child mortality rate^*a*^			*P*-value
			
		Overall	Kilombero	Ulanga	
*Sex*					
Female	5669 (50.9)	27.6	29.8	25.0	0.814
Male	5465 (49.1)	24.7	27.1	21.6	0.795
*Socio-economic status*					
Poorest	2203 (19.8)	31.1	36.5	26.0	0.752
Very poor	2265 (20.3)	26.0	27.5	24.1	0.916
Poor	2281 (20.5)	25.7	29.5	20.6	0.791
Less poor	2239 (20.1)	21.3	21.1	21.6	0.986
Least poor	2146 (19.3)	27.1	28.9	24.5	0.898
*Distance to nearest health facility*					
< 1 km	2793 (25.1)	23.3	25.9	20.7	0.860
1 – 4.9 km	4666 (41.9)	27.2	29.4	24.1	0.819
≥ 5 km	3675 (33.0)	26.9	29.0	24.7	0.865
*Bednet density at household level*^*b*^					
0	1199 (10.8)	28.9	40.4	19.5	0.662
0 – 0.2	2531 (22.7)	27.9	28.9	26.8	0.946
0.2 – 0.3	3426 (30.8)	28.5	30.3	28.5	0.873
0.3 – 0.5	3026 (27.2)	22.3	22.4	22.3	0.996
> 0.5	952 (8.5)	22.6	28.9	13.9	0.794

^*a *^: Mortality rate per 1000 person years.

^*b *^: Number of bednets per person within a 0 m radius around each household.

A simple bivariate analysis showed that bednet density at household level was significantly associated with child mortality (*IRR *= 0.50, *P *= 0.020). There was a tendency for mortality rates to decrease for children living in households with at least 30% bednet density coverage.

The effect of various bednet density measures on child mortality after adjusting for possible confounders is shown in Table 2. Surprisingly, the only measure significantly associated with child mortality was the bednet density at household level (*R*_0_) (*IRR *= 0.53, *P *= 0.037). We noted that the mean bednet density was similar for all radii, whereas the standard deviation tended to become smaller as the radius was increasing.

**Table 2:** Summary of bednet density measures and estimates of the effect of bednet measures on child mortality, adjusted by sex, socio-economic status and distance to the nearest health facility

Bednet density	Mean (St. dev.)	% of households without bednets	IRR^*a*^	95% CI	LRT^*b*^	*P*-value^*c*^
*R*_0_	0.25 (0.15)	0.00	0.53	(0.29,0.97)	4.37	0.037
*R*_50_	0.18 (0.20)	0.09	0.64	(0.40,1.03)	3.48	0.062
*R*_100_	0.24 (0.18)	12.68	1.13	(0.73,1.74)	0.27	0.607
*R*_150_	0.25 (0.13)	13.84	1.18	(0.61,2.30)	0.24	0.623
*R*_200_	0.26 (0.12)	14.44	1.69	(0.79,3.61)	1.79	0.181
*R*_300_	0.26 (0.09)	15.04	2.51	(0.96,6.55)	3.46	0.063
*R*_400_	0.27 (0.08)	15.04	2.10	(0.79,5.59)	2.05	0.153
*R*_500_	0.27 (0.07)	15.18	2.40	(0.70,8.25)	1.89	0.169
*R*_600_	0.27 (0.07)	15.23	2.89	(0.74,11.25)	2.32	0.128

Results obtained by fitting negative binomial models.

^*a *^: IRR:Incidence-rate ratios.

^*b *^: LRT:Likelihood ratio test.

^*c *^: *P *-value based on likelihood ratio test (LRT).

The results of the bivariate and multivariate non-spatial negative binomial models are shown in Table 3. None of the explanatory variables were significantly associated with child mortality, except the fourth wealth quintile. After taking into account the spatial correlation present in the data, the effect of the covariates remained non-significant. However, the confidence intervals became wider, confirming the importance of taking into account spatial correlation when analyzing geographical data [19]. The parameters *σ*^2 ^and *ρ *shown in Table 3 measure the spatial variance and the rate of correlation decay (smoothing parameter), respectively. The estimates of the smoothing parameter *ρ *indicate a low spatial correlation in the child mortality rate data. In fact *ρ *was estimated to be 774.5, which in our exponential setting is translated to a minimum distance for which spatial correlation decrease to 0.05 of only around 0.43 km.

**Table 3:** Results of the association of sex, socio-economic status, bednet density at household level and distance to nearest health facility with child mortality, resulting from the bivariate and multivariate non-spatial models and spatial model

Indicator	Bivariate model		Multivariate model		Spatial model	
	
	IRR^*a*^	95% CI^*b*^	IRR^*a*^	95% CI^*b*^	IRR^*a*^	95% CI^*b*^
*Sex*						
Female	1.0		1.0		1.0	
Male	0.90	(0.75,1.07)	0.89	(0.75,1.06)	0.88	(0.73,1.06)
*Socio-economic status*						
Most poor	1.0		1.0		1.0	
Very poor	0.83	(0.64,1.09)	0.84	(0.65,1.11)	0.87	(0.63,1.21)
Poor	0.82	(0.63,1.08)	0.84	(0.64,1.10)	0.82	(0.64,1.05)
Less poor	0.69	(0.52,0.91)	0.70	(0.53,0.93)	0.68	(0.51,0.94)
Least poor	0.87	(0.67,1.14)	0.90	(0.69,1.18)	0.90	(0.68,1.20)
*Bednet density at household level*						
0	1.0		1.0		1.0	
0 – 0.2	0.96	(0.71,1.32)	0.99	(0.73,1.36)	1.03	(0.83,1.29)
0.2 – 0.3	0.99	(0.73,1.33)	1.02	(0.76,1.39)	1.04	(0.81,1.39)
0.3 – 0.5	0.77	(0.57,1.06)	0.81	(0.59,1.11)	0.84	(0.56,1.13)
> 0.5	0.78	(0.52,1.17)	0.81	(0.54,1.23)	0.76	(0.54,1.24)
*Distance to nearest health facility*	0.80	(0.16,3.96)	0.60	(0.10,3.68)	0.23	(0.03,3.71)
*Spatial parameters*						
*σ*^2^					0.75	(0.35,1.16)
Range (3/*ρ*)^*c*^					0.43	(0.39,0.48)

^*a *^: IRR:Incidence-rate ratios.

^*b *^: Credible intervals.

^*c *^: Spatial correlation is significant (> 5%) within this distance.

Table 4 depicts the effect of different bednet density measures on the mortality of children without any bednet after adjusting for sex, socio-economic status and distance to the nearest facility. The results show no significant association between any bednet density measure and mortality of children without nets, indicating no detectable community effect.

**Table 4:** Estimated effect of bednet measures on mortality of children without nets, adjusted by sex, socioeconomic status and distance to the nearest health facility, obtained by fitting negative binomial models

Bednet density	Incidence risk ratio				*P*-value^*a*^
		
	No bednet	0 – 0.2	0.2 – 0.3	> 0.3	
*R*_50_	1.0	0.88 (0.42,1.83)	0.70 (0.31,1.60)	0.89 (0.42,1.71)	0.935
*R*_100_	1.0	0.64 (0.29,1.42)	1.04 (0.51,2.10)	1.15 (0.57,2.34)	0.519
*R*_150_	1.0	0.82 (0.34,1.98)	0.91 (0.38,2.17)	2.06 (0.91,4.64)	0.077
*R*_200_	1.0	0.74 (0.30,1.79)	0.68 (0.28,1.63)	1.35 (0.57,3.20)	0.303
*R*_300_	1.0	1.18 (0.33,4.16)	1.64 (0.48,5.62)	1.40 (0.38,5.08)	0.713
*R*_400_	1.0	1.40 (0.31,6.28)	1.90 (0.44,8.24)	1.49 (0.32,7.00)	0.809
*R*_500_	1.0	1.97 (0.26,15.15)	2.31 (0.31,17.38)	1.80 (0.22,14.54)	0.732
*R*_600_	1.0	1.22 (0.16,9.33)	1.63 (0.22,12.03)	1.14 (0.14,9.06)	0.811

^*a *^: *P*-value based on likelihood ratio test (LRT).

Pearson's correlation coeffcient between bednet density and bednet usage was 0.83, indicating a strong correlation between the two measures. Hence, the results regarding the bednet density could be extended to bednet usage.

## Conclusion and discussion

We examined the effect of a variety of factors on child mortality in an area of high perennial malaria transmission in southern Tanzania and identified that the density of household bed net ownership was the only factor significantly associated with child mortality reduction. The spatial effects of bednets on all-cause child mortality in an area of high perennial malaria transmission in southern Tanzania have been presented here. The effect of different bednet density measures was estimated after adjusting for possible confounders like sex, socio-economic status and distance to the nearest health facility. We concentrated on all-cause child mortality because in rural Africa it is difficult to assess malaria-specific mortality. Most deaths occur at home and verbal autopsy is the only tool available to determine the cause of mortality. It has been shown [20,21] that this is an inaccurate method to detect malaria, having a low sensitivity and specificity.

Our results indicated an apparent lack of community effect of bednets on childhood mortality. This conclusion is based on the fact that only the bednet density at household level had a significant protective effect on child mortality. When net density within ≥ 50 m was considered, the risk of child mortality increased slightly but the relation was not significant. Our findings contrast with previous studies in Africa, which demonstrated a strong community-wide effect of ITNs on child mortality [8,9]. However, our study differed from the studies mentioned above in a number of ways.

Firstly, the epidemiological studies that demonstrated the mass effect of ITNs on child mortality were all designed as community-trial interventions, ensuring a uniformly high coverage of treated nets in the intervention group, with a control group almost not using any sort of nets. This creates a strong gradient of ITN at the margins use, which allows a good measure of spatial effects. By contrast, net usage, treated or not, was uniformly high in our study area, with the result that any sort of spatial effects would be more difficult to detect unless there would be heterogeneity in coverage, which was not the case.

Secondly, we were not able to distinguish between treated and untreated nets in the field because there is no reliable testing method to do this at present. [17,22] showed that in our study area compliance with insecticide re-treatment is relatively low, with only 32% of the nets having enough insecticide to ensure an entomological impact. Since untreated nets are less effective than treated ones [4,5,23], this had certainly an impact on the analysis by reducing differences between users and non-users. However, despite these limitations, our study showed that mosquito nets still show a protective effect on child mortality.

Lastly, as specific data on bednet use was not available for the whole sample, we created a different measure of the impact of bednets: the "bednet density" defined as the ratio between the number of bednets owned and the number of people living in a specific area. Previous studies in this region showed that on average 2 people sleep under a bednet with an overall bednet use of about 75% [16]. A limitation of our study consists in linking child mortality data across 2 years (2001 – 2003) with data on mosquito net ownership collected at a single time point (2002).

All analyzes of bednets effect on different malaria-related outcomes so far have been based on the assumption of independence between observations. However, household mortality data are spatially correlated due to common exposures. When the spatial correlation present in the data is ignored, the statistical significance of the covariates is overestimated. We could control for that by using a Bayesian geostatistical approach to assess the child mortality-bednet density relation. Bayesian computation implemented via MCMC enabled simultaneous estimation of all model parameters together with their standard errors, a feature that is not available in the maximum likelihood based framework. Fitting geostatistical models for non-Gaussian data requires repeated inversions of the covariance matrix of the spatial random effects. These computations are not feasible when analyzing DSS mortality data collected at very large number of locations. A convolution model for the underlying spatial process has been suggested for handling large spatial data sets. This approach can be further applied for modeling mortality data coming from other DSS sites.

Despite these limitations, our results are consistent with the analysis of ITN' protective efficacy against malaria transmission in Kilombero Valley [24], which predicted little community-level protection for the individuals not using ITNs. The most likely explanations for this were the small proportion of re-treated nets and the insufficient concentration of insecticide present in the bednets. A recently developed model for the transmission of malaria using data collected in Tanzania [7] predicted that modest bednet coverage (35% – 65%) of the entire population, rather than just high-risk groups (pregnant women and young children) is needed to achieve community-wide protection similar to, or greater than, individual protection. Hence, there is clearly a strong case for improving the status of insecticide treatment through the introduction of long-lasting insecticidal nets (LLINs) which are now becoming increasingly available [25] and for the wide-use of ITNs and LLINs by the whole population. We expect that achieving a high coverage with LLINs will result in further substantial reductions of malaria transmission and hence malaria-related mortality and morbidity for both users and non-users.

## Competing interests

The authors declare that they have no competing interests.

## Authors' contributions

LG performed the statistical analysis, contributed to the interpretation of the data and drafted the manuscript. PV has made substantial contribution to the spatial statistical methodology and helped in drafting the manuscript. AT, RN and HG participated in the conception and design of the study, collection of the data and have been involved in revising the manuscript. CL contributed to the conception and design of the study, its coordination, have been involved in the analysis of data and helped to draft the manuscript. All authors read and approved the final manuscript.

## Appendix

Let *Y*_*il *_be the mortality outcome of child *l *at site *s*_*i*_*i *= 1, ... , *n *taking value 1 if the child is dead and 0 otherwise. We assume that *Y*_*il *_arises from a negative binomial distribution, that is *Y*_*il*_*~NegBin*(*p*_*il*_, *r*), where *p*_*il *_is the probability that child *l *at location *s*_*i *_is dead and *r *is the parameter that quantifies the amount of extra Poisson variation. To account for spatial variation in the data, location-specific random effects were integrated in the negative binomial model. The probability *p*_*il*_, is modeled as $p_{il}=\frac{r}{r+z_{il}}$, with *log*(*z*_*il*_) = *log*(*pyrs*_*il*_) + $X_{il}^{T}\beta$ + *ϕ*_*i*_, where *X*_*il *_is the vector of associated covariates, *β *are the regression coeffcients and *ϕ*_*i*_'s are the spatial random effects. *pyrs*_*il *_represents the number of person-years corresponding to child *l *at location *s*_*i *_and *log*(*pyrs*_*il*_) is considered as covariate with regression coeffcient fixed to 1 and is referred as offset.

The standard approach to model the spatial dependence is to assume that the covariance of *ϕ*_*i*_'s at every two locations *s*_*i *_and *s*_*j *_decreases with their distance *d*_*ij*_, that is Σ_*ij *_= *σ*^2^*f *(*d*_*ij*_; *ρ*) with *f*(*d*_*ij*_; *ρ*) = *exp*(*-d*_*ij*_*ρ*), where *ρ *> 0 is a smoothing parameter that controls the rate of correlation decay with increasing distance and *σ*^2 ^quantifies the amount of spatially structured variation. Estimation of the location-specific random effects and of the spatial parameters requires repeated inversions of the covariance matrix Σ. Due to the large number of locations in our dataset (7, 403), matrix inversion is computationally intensive and is not feasible within practical time constrains. To overcome this issue we develop a convolution model for the underlying spatial process. In particular, we choose a small number of locations *t*_*k*_, *k *= 1, ... , *K *over the study region, assume a stationary spatial process *ω*_*k *_over these locations and we model the spatial random effect *ϕ*_*i *_at each data location *s*_*i *_as a weighted sum of the fixed location stationary processes. That is, $\phi_{i}=\sum_{k=1}^{K}a(i,k)\omega_{k}$, where the weights *a*(*i, k*) are decreasing functions of the distance between data location *s*_*i *_and the fixed location *t*_*k *_and *ω*_*k*_*~N *(0, Σ_*k*_), with (Σ_*k*_)_*hl *_= *σ*^2^*exp*(*-d*_*hl*_*ρ*), where *d*_*hl *_is the distance between the fixed locations *t*_*h *_and *t*_*l*_. This approach avoids the inversion of the large covariance matrix *nxn*, reducing the problem to the inversion of a much smaller size matrix *KxK*. For this specific analysis we have chosen *K *= 200.

For the correlation function chosen, the minimum distance for which spatial correlation between locations is below 5% is 3/*ρ *(range). The above specification of spatial correlation is isotropic, assuming that correlation is the same in all directions.

Following a Bayesian model specification, we adopt prior distributions for the model parameters as follows: non-informative uniform prior distributions for the regression coeffcients *β*, inverse gamma prior distribution for *σ*^2 ^and gamma prior distribution for the decay parameter *ρ *and the over-dispersion parameter *r*.

We estimate the model parameters using Markov chain Monte Carlo simulation. In particular we implemented Gibbs sampler (Gelfand and Smith, 1990), which requires simulating from the full conditional distributions of all parameters iteratively until convergence. The full conditional distribution of *σ*^2 ^is inverse gamma distribution and it is straightforward to simulate from. The conditional posterior distribution of *β*, *ρ*, and *r *do not have known forms. We simulate from these distributions using the Metropolis algorithm with a Normal proposal distribution having the mean equal to the parameter estimate from the previous Gibbs iteration and the variance equal to a fixed number, iteratively adapted to optimize the acceptance rates. We have run a five-chain sampler with a burn-in of 10, 000 iterations and we assessed the convergence by inspection of ergodic averages of selected model parameters after 200, 000 iterations.

## Pre-publication history

The pre-publication history for this paper can be accessed here:


